# Developing Two Rapid Protein Extraction Methods Using Focused-Ultrasonication and Zirconia-Silica Beads for Filamentous Fungi Identification by MALDI-TOF MS

**DOI:** 10.3389/fcimb.2021.687240

**Published:** 2021-07-06

**Authors:** Ya-Ting Ning, Wen-Hang Yang, Wei Zhang, Meng Xiao, Yao Wang, Jing-Jia Zhang, Ge Zhang, Si-Meng Duan, Ai-Ying Dong, Da-Wen Guo, Gui-Ling Zou, Hai-Nan Wen, Yan-Yan Guo, Li-Ping Chen, Miao Chai, Jing-Dong He, Qiong Duan, Li-Xia Zhang, Li Zhang, Ying-Chun Xu

**Affiliations:** ^1^ Department of Clinical Laboratory, State Key Laboratory of Complex Severe and Rare Diseases, Peking Union Medical College Hospital, Chinese Academy of Medical Sciences and Peking Union Medical College, Beijing, China; ^2^ Graduate School, Chinese Academy of Medical Sciences and Peking Union Medical College, Beijing, China; ^3^ Beijing Key Laboratory for Mechanisms Research and Precision Diagnosis of Invasive Fungal Diseases, Beijing, China; ^4^ Clinical Microbiology Laboratory, The First Affiliated Hospital of Hebei North University, Zhangjiakou, China; ^5^ Department of Clinical Laboratory, North China University of Science and Technology Affiliated Hospital, Tangshan, China; ^6^ Department of Clinical Laboratory, The First Affiliated Hospital of Harbin Medical University, Harbin, China; ^7^ Department of Clinical Laboratory, The Fourth Affiliated Hospital of Harbin Medical University, Harbin, China; ^8^ Department of Laboratory, The Affiliated Hospital of Chengde Medical University, Chengde, China; ^9^ Department of Clinical Laboratory, Tangshan Worker’s Hospital, Tangshan, China; ^10^ Department of Laboratory Medicine, Mudanjiang First People’s Hospital, Heilongjiang, China; ^11^ Department of Clinical Laboratory, The First Hospital of Harbin, Harbin, China; ^12^ Department of Clinical Laboratory, Tianjin Chest Hospital, Tianjin, China; ^13^ Department of Clinical Laboratory, Jinling Province People’s Hospital, Jinling, China; ^14^ Department of Clinical Laboratory, Shanxi Provincial People’s Hospital, Taiyuan, China

**Keywords:** filamentous fungi, MALDI-TOF MS, protein extraction, sample processing, zirconia-silica beads, focused-ultrasonication, in-house library

## Abstract

Filamentous fungi identification by Matrix-assisted laser desorption ionization time-of-flight mass spectrometry (MALDI-TOF MS) has been challenging due to the lack of simple and rapid protein extraction methods and insufficient species coverage in the database. In this study, we created two rapid protein extraction methods for filamentous fungi: a one-step zirconia-silica beads method (ZSB) and a focused-ultrasonication method (FUS). The identification accuracy of two methods were evaluated with the VITEK MS, as well as number of spectra peaks and signal-to-noise ratio (S/N) with M-Discover 100 MALDI-TOF MS compared to the routine method. The better method was applied to build a filamentous fungi in-house spectra library for the M-Discover 100 MS, and then another one and routine method were performed in parallel to verify the accuracy and commonality of the in-house library. Using the two optimized methods, the dedicated operating time before MALDI-TOF MS analysis was reduced from 30 min to 7 (ZSB) or 5 (FUS) min per sample, with only a few seconds added for each additional strain. And both two methods identified isolates from most mold types equal to or better than the routine method, and the total correct identification rate using VITEK MS was 79.67, 76.42, and 76.42%, respectively. On the other hand, the two rapid methods generally achieved higher maximum and minimum S/N ratios with these isolates tested as compared to the routine method. Besides, the ZSB method produced overall mean of maximum and minimum S/N ratio higher than that by FUS. An in-house library of M-Discover MS was successfully built from 135 isolates from 42 species belonging to 18 genera using the ZSB method. Analysis of 467 isolates resulted in 97.22% correctly identified isolates to the species level by the ZSB method *versus* 95.50% by the routine method. The two novel methods are time- and cost-effective and allow efficient identification of filamentous fungi while providing a simplified procedure to build an in-house library. Thus, more clinical laboratories may consider adopting MALDI-TOF MS for filamentous fungi identification in the future.

## Introduction

In recent years, fungi have come to pose a serious threat to immunocompromised patients with leukemia, AIDS, or receiving chemotherapy intervention, etc. (Enoch et al., 2006; Benedict et al., 2017; Bongomin et al., 2017). Even though *Candida* remains the leading invasive fungi pathogen, infections due to filamentous fungi are gradually rising with high mortality (Enoch et al., 2006; Liao et al., 2013; Benedict et al., 2017; Bongomin et al., 2017). However, appropriate treatment often varies by species, thus making rapid identification essential for accurate diagnosis and better outcomes (Brown et al., 2012). Conventional identification methods of filamentous fungi based on morphological traits are time-consuming and require extensive expertise training (Larone, 2011). Moreover, less common or non-sporulating molds are difficult to identify, and phylogenetically related species with similar morphological features are challenging to discriminate (Kozel and Wickes, 2014; Li et al., 2017; Luethy and Zelazny, 2018; Wickes and Wiederhold, 2018). Molecular identification is the gold standard method to identify the above strains, but is relatively expensive and requires specialized equipment which limits its routine use in clinical laboratories (Balajee et al., 2007; Samson et al., 2014; Wickes and Wiederhold, 2018).

Matrix-assisted laser desorption/ionization-time of flight mass spectrometry (MALDI-TOF MS) has emerged as a cost-effective and rapid alternative for mycobacterial, bacterial, and yeast identification (Seng et al., 2009; Fraser et al., 2016; Rodriguez-Sanchez et al., 2016). Currently, its use for the identification of filamentous fungi has gradually begun to be implemented in clinical microbiology laboratories, but has been hampered by commercial databases with limited coverage of filamentous fungi taxa and challenges of protein extraction to obtain good quality mass spectra for analysis (Welham et al., 2000; Cassagne et al., 2016; Santos et al., 2017). Building an in-house database that contains local or less common isolates is the most optimal way to overcome the deficiencies of commercial databases (Zvezdanova et al., 2019). At present, several in-house databases in the Bruker MALDI Biotyper (Bruker Daltonics, Germany) have been developed by laboratories, significantly increasing species-assignment of filamentous fungi (De Carolis et al., 2012; Becker et al., 2014; Gautier et al., 2014; Schulthess et al., 2014; Luethy and Zelazny, 2018; Zvezdanova et al., 2019).

Protein extraction is the most critical step of filamentous fungi identification by MALDI-TOF MS, but this process faces challenges due to the robust chitinous cell wall of filamentous fungi and necessitating protein extraction via a process usually initialized by cell homogenization (Shapaval et al., 2017; Krishnaswamy et al., 2019). Moreover, routine protein extraction methods in the manufacturer’s instructions and laboratory-developed procedures involve multiple steps (i.e. wash, inactivation, chemical extraction) and require 30 min to over an hour to perform (Chakrabarti et al., 2015; Cassagne et al., 2016). Thus, a simpler and more rapid procedure for protein extraction is urgently needed to permit routine use of MALDI-TOF MS for filamentous fungi identification in clinical laboratories. Ultrasound disruption is a common mechanical cell homogenization method based on high shear force, applied successfully in MALDI-TOF MS for mycobacterial identification and LC-MS/MS (Klimek-Ochab et al., 2011; Adams et al., 2016). Adaptive focused acoustics *via* concentrated bursts of higher-frequency ultrasonic energy allows for rapid disruption of the cell wall and concomitant protein extraction into the extraction solution within minutes (Li et al., 2015; Adams et al., 2016). Another mechanical method is bead milling, such as through the use of zirconia-silica beads and zirconium beads (Krishnaswamy et al., 2019). Proteins are released by the action of circulating beads dispersed in the fluid (Doucha and Livansky, 2008; Klimek-Ochab et al., 2011).

To simplify and expedite the sample processing before the identification of filamentous fungi isolates by MALDI-TOF MS, we created two rapid protein extraction procedures: the one-step zirconia-silica beads (ZSB) method and the focused-ultrasonication method (FUS). In this study, we investigated the identification accuracy of two rapid sample processing methods in the VITEK MALDI-TOF MS system (bioMérieux, Marcy-l’Étoile, France), as well as number of mass peaks and signal-to-noise (S/N) ratio in M-Discover 100 MALDI-TOF MS (Zhuhai Meihua Medical Technology Co., Ltd., China) *versus* the routine method. Then according to the results, applied the better method as a means to build a filamentous fungi in-house spectra library for the M-Discover 100 MS. In addition, we evaluated the accuracy and commonality of the in-house library using the new method consisting of building the database and the routine method.

## Methods and Materials

### Isolates and Species Identification

A total of 602 non-duplicate mold isolates recovered from various clinical specimens of patients were under the China Hospital Invasive Fungal Surveillance Net–North China Program. Isolates were cultured on Sabouraud Dextrose Agar (SDA) plates (Becton Dickinson Microbiology Systems, Sparks, MD, USA) and incubated at 28^◦^C for 2 to 5 days, and mycelia were collected for genomic DNA extraction. The internal transcribed spacer region was carried out as the primary sequencing gene for species level identification (Zvezdanova et al., 2019). The β-tubulin gene was employed additionally for the *Scedosporium/Pseudallescheria* spp., as well as the translation elongation factor 1-α gene for the *Fusarium* spp. (Gilgado et al., 2008; Wang et al., 2011). Sequencing data was analyzed using the National Center for Biotechnology Information (NCBI) or Mycobank database, and the results were accepted if homology >98% with >95% query coverage. One hundred twenty-three isolates belonging to 13 mold genera and 29 species were analyzed by VITEK MS to evaluate three protein extracting methods (**Table 1**). Another 135 clinical isolates were included in the in-house library of M-Discover 100 MS (**Table 2**); the remaining 467 isolates were analyzed using the in-house library coupled with the rapid method and routine method (**Table 3**).

**Table 1:** Identification of 123 clinical filamentous fungi isolates by VITEK MS using the routine method in comparison with two rapid methods.

Identification by DNA sequencing analysis	Reference spectra	Number	Routine method			ZSB method			FUS method		
			Correct ID	Incomplete ID	No ID	Correct ID	Incomplete ID	No ID	Correct ID	Incomplete ID	No ID
***Aspergillus***	**total**	**66**	**64**	**0**	**2**	**64**	**0**	**2**	**63**	**0**	**3**
A. flavus*	√	10	10	0	0	10	0	0	10	0	0
* A. fumigatus*	√	10	9	0	1	10	0	0	10	0	0
* A. lentulus*	√	3	3	0	0	3	0	0	3	0	0
* A. luchuensis*	×	1	0	0	1	0	0	1	0	0	1
* A. nidulans*	√	10	10	0	0	10	0	0	8	0	2
* A. niger*	√	10	10	0	0	10	0	0	10	0	0
* A. sydowii*	√	1	1	0	0	1	0	0	1	0	0
* A. terreus*	√	10	10	0	0	10	0	0	10	0	0
A. tubingensis*	√	10	10	0	0	9	0	1	10	0	0
A. ustus*	√	1	1	0	0	1	0	0	1	0	0
***Fusarium***	**total**	**22**	**13**	**6**	**3**	**16**	**5**	**1**	**14**	**6**	**2**
* F. incarnatum*	×	1	0	1 (*Fch* complex)	0	0	0	1	0	0	1
* F. proliferatum*	√	6	1	2 (*Fve/pr*), 1 (*Fve*)	2	2	4 (*F. ve*)	0	1	3 (*Fve*), 1 (*Fve/pr*)	1
* F. solani*	√	9	9	0	0	9	0	0	9	0	0
* F. verticillioides*	√	6	3	1 (*F. ve/pr*), 1 (*F. pr*)	1	5	1 (*F. ve/pr*)	0	4	2 (*Fve/pr*)	0
***Penicillium***	**total**	**12**	**2**	**0**	**10**	**2**	**0**	**10**	**1**	**0**	**11**
* P. chrysogenum*	√	1	1	0	0	1	0	0	0	0	1
* P. citrinum*	√	1	1	0	0	1	0	0	1	0	0
* P. oxalicum*	×	10	0	0	10	0	0	10	0	0	10
***Scedosporium***	**total**	**5**	**3**	**0**	**2**	**4**	**0**	**1**	**4**	**0**	**1**
* S. apiospermum*	√	2	1	0	1	2	0	0	2	0	0
* S. aurantiacum*	×	1	0	0	1	0	0	1	0	0	1
* S. boydii*	√	2	2#	0	0	2#	0	0	2#	0	0
**Others**	**total**	**18**	**12**	**0**	**6**	**12**	**0**	**6**	**13**	**0**	**5**
* Alternaria alternata*	√	3	1	0	2	1	0	2	2	0	1
* Beauveria bassiana*	√	1	1	0	0	0	0	1	0	0	1
Geotrichum candidum*	√	3	2	0	1	3	0	0	3	0	0
* Mucor circinelloides*	√	1	1	0	0	1	0	0	1	0	0
* Rhizopus oryzae*	√	2	2	0	0	2	0	0	2	0	0
* Scopulariopsis brevicaulis*	×	1	0	0	1	0	0	1	0	0	1
* Sporothrix schenckii*	√	1	1	0	0	1	0	0	1	0	0
* Syncephalastrum racemosum*	×	2	0	0	2	0	0	2	0	0	2
* Trichoderma longibrachiatum*	√	4	4	0	0	4	0	0	4	0	0
**Total**		**123**	**94**	**6**	**23**	**98**	**5**	**20**	**95**	**6**	**22**

*According to the specification of database v3.2, the proteomes of some species are so similar that it is difficult for VITEK MS to distinguish, such as A. flavus and A. oryzae, A. calidoustus and A. ustus, Geotrichum candidum and Geotrichum klebahnii, as well as A. tubingensis which shows “A. niger complex”. Those results were all considered as “correct-ID”.

# VITEK MS identified S. boydii (Pseudallescheria boydii’s asexual stage) as Pseudallescheria boydii. These results were considered as correct.

ID, identification; Fch, F. chlamydosporum; Fve, F. verticillioides; Fpr, F. proliferatum; Fve/pr, F. verticillioides/proliferatum.

**Table 2:** List of isolates included in the in-house library of M-Discover 100 MS.

Identification by DNA sequencing analysis	Number of isolates
***Aspergillus***	
* A. fumigatus*	15
* A. insuetus*	2
* A. japonicus*	1
* A. lentulus*	1
* A. luchuensis*	3
* A. nidulans*	10
* A. niger*	15
* A. oryzae*	4
* A. pseudoglaucus*	1
* A. ruber*	1
* A. sydowii*	8
* A. tamarii*	3
* A. terreus*	12
* A. tubingensis*	15
* A. uvarum*	1
***Penicillium***	
* P. chermesinum*	1
* P. citrinum*	2
* P. oxalicum*	7
***Scedosporium***	
* S. apiospermum*	1
* S. aurantiacum*	1
* S. boydii*	1
***Trichoderma***	
* T. longibrachiatum*	1
* T. asahii*	2
* T. coremiiforme*	1
* T. japonicum*	1
**Others**	
* Alternaria alternata*	1
* Arthrinium* spp.	1
* Beauveria bassiana*	1
* Doratomyces* spp.	1
* Exophiala dermatitidis*	2
* Geotrichum candidum*	2
* Monascus purpureus*	1
* Mucor circinelloides*	3
* Paecilomyces variotii*	1
* Phanerochaete chrysosporium*	1
* Rhizomucor pusillus*	1
* Rhizopus microsporus*	4
* Rhizopus oryzae*	1
* Scopulariopsis* spp.	1
* Syncephalastrum racemosum*	2
* Talaromyces funiculosus*	1
* Talaromyces stollii*	1
**Total**	**135**

**Table 3:** 467 clinical filamentous fungi isolates identified by M-Discover 100 MS with the in-house library using the ZSB method in comparison with the routine method.

Identification by DNA sequencing analysis	Number	ZSB method								Routine method							
		Correct ID to Species Level				Only Correct ID to Genus Level		Mis ID		Correct ID to Species Level				Only Correct ID to Genus Level		Mis ID	
		Subtotal	≥90	90–60	≤60	Number	score	Number	score	Subtotal	≥90	90–60	≤60	Number	score	Number	score
***Aspergillus***																	
* A. flavus/oryzae*	63	63	55	8	0	0	–	0	–	63	46	15	2	0	–	0	–
* A. fumigatus*	184	184	180	4	0	0	–	0	–	183	179	3	1	1	–	0	–
* A. lentulus*	2	1	1	0	0	1	≤60	0	–	1	1	0	0	1	–	0	–
* A. luchuensis*	1	1	1	0	0	0	–	0	–	1	1	0	0	0	–	0	–
* A. nidulans*	20	20	19	1	0	0	–	0	–	20	17	3	0	0	–	0	–
* A. niger*	58	58	58	0	0	0	–	0	–	58	53	5	0	0	–	0	–
* A. sydowii*	1	1	0	1	0	0	–	0	–	1	0	1	0	0	–	0	–
* A. terreus*	32	32	27	5	0	0	–	0	–	32	27	5	0	0	–	0	–
* A. tubingensis*	36	35	35	0	0	1	≤60	0	–	35	32	3	0	1	≤60	0	–
* A. ustus*	1	1	1	0	0	0	–	0	–	1	1	0	0	0	–	0	–
*** Subtotal***	**398**	**396**	**377**	**19**	**0**	**2**	**-**	**0**	**-**	**395**	**357**	**35**	**3**	**3**	**-**	**0**	**-**
***Fusarium***																	
* F. moniliforme*	5	4	3	1	0	1	90–60	0	–	4	2	2	0	1	90–60	0	–
* F. proliferatum*	6	6	0	6	0	0	–	0	–	4	0	2	2	1	90–60	1	≤60
* F. solani*	9	7	4	3	0	0	–	2	≤60	8	0	5	3	0	–	1	≤60
* F. verticillioides*	6	0	0	0	0	6	3(>90) 3(90–60)	0	–	0	0	0	0	6	2(>90) 3 (90–60) 1 (≤60)	0	–
*** Subtotal***	**26**	**17**	**7**	**10**	**0**	**7**	**0**	**2**		**16**	**2**	**9**	**5**	**8**	**-**	**2**	**-**
***Penicillium***																	
* P. chrysogenum*	1	1	0	0	1	0	–	0	–	1	0	0	1	0	–	0	–
* P. citrinum*	1	1	1	0	0	0	–	0	–	1	1	0	0	0	–	0	–
* P. oxalicum*	16	16	16	0	0	0	–	0	–	16	15	1	0	0	–	0	–
** Subtotal**	**18**	**18**	**17**		**1**	**0**	**-**	**0**	**-**	**18**	**16**	**1**	**1**	**0**	**-**	**0**	**-**
***Scedosporium***																	
* S. apiospermum*	2	2	1	1	0	0	–	0	–	2	1	1	0	0	–	0	–
* S. aurantiacum*	1	1	1	0	0	0	–	0	–	1	1	0	0	0	–	0	–
* S. boydii*	2	2	2	0	0	0	–	0	–	2	2	0	0	0	–	0	–
** Subtotal**	**5**	**5**	**4**	**1**	**0**	**0**	**-**	**0**	**-**	**5**	**4**	**1**	**0**	**0**	**-**	**0**	**-**
**Others**																	
* Alternaria alternata*	3	3	3	0	0	0	–	0	–	2	0	2	0	0	–	1	≤60
* Beauveria bassiana*	1	1	0	1	0	0	–	0	–	0	0	0	0	0	–	1	≤60
* Geotrichum candidum*	3	3	3	0	0	0	–	0	–	3	3	0	0	0	–	0	–
* Mucor circinelloides*	1	1	1	0	0	0	–	0	–	1	1	0	0	0	–	0	–
* Rhizopus oryzae*	2	0	0	0	0	1	≤60	1	≤60	0	0	0	0	0	–	2	≤60
* Scopulariopsis brevicaulis*	1	1	1	0	0	0	–	0	–	1	1	0	0	0	–	0	–
* Sporothrix schenckii*	1	1	0	0	1	0	–	0	–	1	0	0	1	0	–	0	–
* Syncephalastrum racemosum*	2	2	1		1	0	–	0	–	1	1	0	0	0	–	1	≤60
* Trichoderma longibrachiatum*	4	4	4	0	0	0	–	0	–	1	1	0	0	3	90–60		–
* Trichosporon asahii*	2	2	1	1	0	0	–	0	–	2	2	0	0	0	–	0	–
** Subtotal**	**20**	**18**	**14**	**2**	**2**	**1**	**-**	**1**	**-**	**12**	**9**	**2**	**1**	**3**	**-**	**5**	**-**
**Total**	**467**	**454**	**419**	**32**	**3**	**10**	**-**	**3**	**-**	**446**	**388**	**48**	**10**	**14**	**-**	**7**	**-**

### Protein Extraction

#### The Routine Three-Step Method

Isolates were inoculated on SDA plates at 28^◦^C for 3 days. The routine method for protein preparation was performed according to the manufacturer’s instructions as described by Li et al. (Li et al., 2017). Briefly, one to two colonies (~OD_600_ of 2.0) were mixed with 900 µl ethanol and 300 µl distilled water, followed by centrifugation for 3 min at 13,800 g. The pellet was dried at room temperature (RT) for 5 min, and then re-suspended in 50–80 µl of 70% formic acid (FA). After an incubation of 5 min at RT, an equal volume of acetonitrile was added. Samples were incubated again at RT for 5 min and subsequently centrifuged at 13,800 g for 1 min.

#### One-Step Zirconia-Silica Beads Method (ZSB)

Rapid extraction of protein using the ZSB method was performed using the same cultures as the routine method. Approximately 1–2 cm^2^ pieces of mold were removed from the agar and added to a 1.5 ml tube containing 30 μl of zirconia-silica beads with a diameter of 0.5 mm and 60 ul extraction solution (consisting of 30 ul acetonitrile and 30 ul FA). The tubes were vortexed for 5 min at RT, and then centrifuged for 1 min at 13,800 g.

#### Focused-Ultrasonication Method (FUS)

One to two colonies were added to a microtube containing 80 ul of extraction solution, and then processed in a precooled focused-ultrasonicator (Longlight Technology Co., Ltd, China) under the following conditions: pulse period of 500, pulse width of 250, running power of 100, running time of 60 s, and water temperature at 15^◦^C.

### Evaluation of Protein Extraction Methods

Two novel methods of protein extraction were evaluated in parallel with the routine method. One microliter of supernatant after treatment was transferred to the target plate and allowed to dry at RT before being overlaid with 1 ul of matrix solution (ɑ-cyano-4-hydroxy-cinnamic acid). The acquisition and analysis of mass spectra were performed by VITEK MALDI-TOF MS using the Vitek MS database (MS-ID version v3.2). The results were interpreted referred the manufacturer’s instructions. An isolate was considered correctly identified with an acceptable confidence value of 99.9%. Samples were analyzed in duplicates and repeated when there were discrepancies and isolates exhibited discrepant identification results. Results were compared with the sequencing-based identification results and grouped into four categories: a) correct identification: identical to sequencing results, b) incomplete identification (Incomplete ID): either only the genus level was correctly identified or more than one species was proposed and one was correct, c) misidentification (Mis-ID): none of the proposed species were correct, or d) no identification (no-ID).

Spectra were validated with 123 strains by M-Discover 100 MS. The number of peaks and S/N ratios were determined by using the program provided by M-Discover. The maximum (or minimum) S/N ratio is defined as the height of the highest (or lowest) mass peak above its baseline relative to the standard deviation of the noise.

### In-House Database Construction and Clinical Isolates Identification

According to the evaluation results, we selected the more efficient method to build an in-house library for M-Discover 100 MS. One hundred thirty-five isolates were included in the in-house spectra library (**Table 2**) after following the manufacturer’s instructions. Freshly prepared isolates were processed and spotted onto eight-well positions on the target plate. Each position was read three times. After excluding the spectra that were obviously abnormal with others, 20 to 24 replica spectra of each strain were added to the in-house library. To verify the accuracy and commonality of the in-house library, the remaining 467 isolates were analyzed. Following the instructions of M-Discover 100 MS, identification scores of ≥90 indicated species-level identification, scores of 60–90 indicated genus-level identification, and scores of ≤60 were considered as “not reliable” (NRI). If isolates exhibited discrepant identification results or produced low matches by M-Discover 100 MS analysis and sequencing analysis, identification by M-Discover 100 MS analysis for the isolates was repeated. In this study, results were compared at the species and genus level with those obtained by sequencing regardless of score values, and grouped into three categories: a) correct identification to species level, b) only correct identification to genus level, c) misidentification.

### Statistical Analysis

Comparison for the identification rates of three protein extraction methods was performed using GraphPad Prism software. This software was also used to compare scores, the peak number, and S/N ratios between two groups *via* a paired t-test. *P* < 0.05 indicated a statistically significant difference (*p values < 0.05, **p values < 0.01, ***p values < 0.005, ****p values < 0.001).

## Results

### Comparison of Identification Performance in VITEK MS

**Table 1** demonstrates the performance of the VITEK MS system for identifying 123 filamentous fungi clinical isolates using the routine and two rapid methods. Among isolates that underwent repeat testing due to discrepancies with the sequencing results, the repeat and original results were consistent. Testing of clinical isolates with two rapid methods revealed significant time savings compared to the routine method. Following the routine procedure, each filamentous fungi isolate required at least 30 min of sample processing, while the ZSB and FUS method reduced the dedicated operating time to 7 or 5 min per sample, respectively, with only a few seconds added for each additional strain.

Applying the routine protein extraction method recommended by the manufacturer, VITEK MS correctly identified (species-level identification) 94 (76.42%) isolates, whereas 98 (79.67%) and 95 (76.42%) isolates by ZSB and FUS method, respectively. “Incomplete ID” results were produced by the routine and FUS methods for 6 (4.88%) isolates, and 5 isolates (4.07%) by the ZSB method, which all belonged to the *Fusarium* spp. None of isolates showed a “Mis-ID” result using all methods, while 23 (18.70%), 20 (16.26%) and 22 (17.89%) isolates had “no-ID” results by the routine, ZSB, and FUS methods. Of these “no-ID” isolates, 16 isolates belonging to six species were due to no reference spectra available in v3.2 database, except one *F. incarnatum* that was correctly identified to genus level as “*F. chlamydosporum complex*” by the routine method.

After excluding the isolates that lacked reference spectra, the success rate of identification by VITEK MS applying the routine, ZSB, or FUS method was 92.52% (99/107) *vs* 96.26% (103/107) *vs* 94.39% (94/107), and the accuracy was up to 100% for *Aspergillus*, *Penicillium*, *Scedosporium*, and other spp., except for *Fusarium* spp. with 72.22% (13/18), 76.19% (16/21), and 70.00% (14/20), respectively by three methods. The remaining 8 (8.75%), 4 (2.50%), and 6 (6.25%) isolates had “no-ID”, respectively. Upon further analysis, for the *Fusarium* spp., the VITEK MS showed good ability to identify *F. solani* (100% accuracy rate by three methods), but could not accurately identify *F. proliferatum* and *F. verticillioides*. The identification ability for *F. proliferatum* and *F. verticillioides* by the ZSB method was 33.33% (1/6) and 83.33% (5/6) with none showing as “no-ID”, followed by the FUS method showing 16.67% (1/6) and 66.67% (4/6) with only one *F. proliferatum* as “no-ID”, and 16.67% (1/6) and 33.33% (2/6), with two *F. proliferatum* and one *F. verticillioides* showing as “no-ID” by the routine method. VITEK MS was unable to distinguish between the remaining *F. proliferatum* and *F. verticillioides*.

Overall, both ZSB and FUS methods identified isolates from each mold type equal to or better than the routine method without statistically significant differences.

### Comparison of Spectral Characteristics in M-Discover 100 MS

**Table S1** shows the number of peaks per strain by three methods. Overall, the number of peaks performed by the routine method was significantly higher than that of two rapid methods (*P* < 0.0001). Among the 123 isolates, the peak number of 28 strains extracted by ZSB was more than that extracted by routine method, and 6 strains were the same, mainly distributed in *Aspergillus terreus* and *A. tubingensis*. For FUS, 32 strains more than, while 12 strains equal to that of routine method, mainly distributed in *A. flavus*, *A. terreus*, and *A. tubingensis*. And the results of FUS were significantly higher than ZSB (*P* = 0.0129).

**Figure 1:**
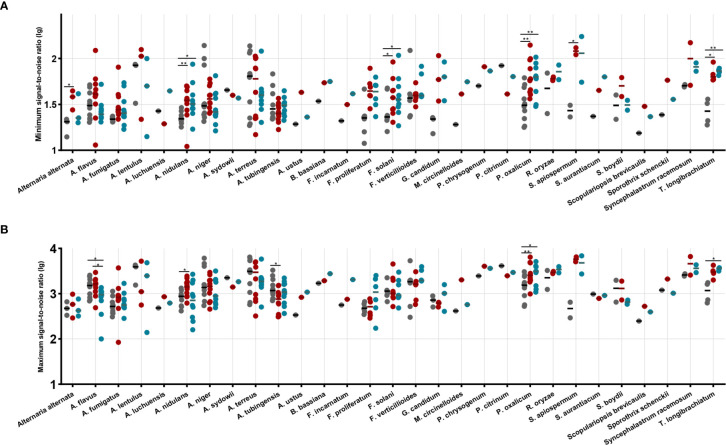
The maximum signal-to-noise ratio **(A)** and the minimum signal-to-noise ratio **(B)** by species and by method performed. Each dot represents the signal-to-noise ratio (lg) for each isolate identification achieved by routine (black dots), ZSB (red dots), and FUS (blue dots) methods. Horizontal bars represent the mean ratio (lg) achieved by each method for each species including at least two isolates. A paired t-test was performed to analyze differences between each two groups, and statistical significance was defined by *p* values less than 0.05 (*p < 0.05, **p < 0.01).

The minimum and maximum S/N ratios for each isolate are showed in **Figure 1**. Overall comparing with routine method, significant increase in minimum S/N ratios was noted for ZSB (*P* < 0.0001) and FUS (*P* = 0.0041) (**Figure 1A**). The mean ratio for each species that had two clinical isolates was higher for 16/18 species by ZSB and for 14/18 species by the FUS method, lower than the routine method for *A. terreus* and *A. tubingensis* by ZSB, and *A. flavus*, *A. lentulus*, *A. niger*, *A. terreus* by FUS. In addition, statistically higher minimum S/N ratios were obtained for *A. nidulans*, *F. solani*, *P. oxalicum*, and *T. longibrachiatum* by both two rapid methods, and *Alternaria alternata* and *S. apiospermum* only by ZSB as compared to the routine method.

As observed for the maximum S/N ratios, the two rapid method achieved higher overall mean ratio with the 123 isolates (1,784.1, 1,678.4, and 1,514.4 for the ZSB, FUS, routine method, respectively). Statistically higher maximum S/N ratios were obtained for *A. nidulans*, *A. tubingensis*, and *P. oxalicum* by ZSB, while for *A. flavus*, *P. oxalicum*, and *T. longibrachiatum* by FUS as compared to the routine method. The ZSB method produced overall mean ratio higher than that by FUS, and showed superior performance for *A. flavus* compared to FUS (**Figure 1B**).

### Identification of Filamentous Fungi by In-House Library of M-Discover 100 MS Built by ZSB

According to the above evaluation results, we selected the ZSB method to build an in-house library for M-Discover 100 MS. From the 135 strains distributed by 42 species and 18 genera (**Table 2**), 2,960 reference spectra were successfully created.

Of the 467 clinical isolates tested, the implementation of the ZSB protein extraction method allowed the correct identification of 99.50% *Aspergillus*, 65.38% *Fusarium* (absent in the in-house library), 100% *Penicillium/Scedosporium*, 90% other molds, and 454/467 (97.22%) total clinical isolates by M-Discover 100 MS at the species level using the in-house library regardless of score values (**Table 3**). Among these 454 isolates, the scores were between 55.76 and 96.90 (median 92.6). A score of ≥90 was obtained for 419 isolates (92.30%), a score of 60–90 for 32 isolates (7.05%), and a score of ≤60 for 3 isolates (0.66%). Ten isolates (2.20%) were identified at the genus level. All *F. verticillioide* (n = 6) and one *F. moniliforme* were identified as *F. moniliforme* (n = 4)/*F. proliferatum* (n = 2, scores 60–90) and *F. proliferatum*; one *A. lentulus*, one *A. tubingensis*, and one *Rhizopus oryzae* were unreliably identified as *A. uvarum* (score 40.94), *A. niger* (score 52.25), and *R. baikonurensis* (score 43.41). In addition, two *F. solani* and one *R. oryzae* were completely misidentified as *Mycobacterium immunogenum/M. malmoense* and *Nocardia cyriacigeorgicascore* (all scores ≤60).

In contrast, when the routine method was coupled with the in-house library, the correct species-level identification rate was 99.74%%, 61.54%%, 100%, 60%, and 95.50% (n = 446, scores ≥90 for 388 isolates, 60–90 for 48 isolates, and ≤60 for 10 isolates) for *Aspergillus*, *Fusarium*, *Penicillium/Scedosporium*, other molds, and total isolates, respectively, and 3.00% (n = 14) to the genus level, thus demonstrating a 1.50% (n = 7, and all with NRI) discrepancy compared to molecular identification. There was no statistically significant difference between the routine and ZSB methods using the in-house library. However, the ability to identify the *Fusarium* spp. was relatively weak. Among 16/26 (61.54%) correctly identified *Fusarium* spp. isolates, only two *F. moniliforme* were completely identified with a high confidence level (score ≥90) and up to five isolates with NRI. In addition, compared to the ZSB method, the routine method was inferior for some rare species, including *Alternaria alternata*, *Beauveria bassiana*, *Rhizopus oryzae*, *Syncephalastrum racemosum.*

**Figure 2:**
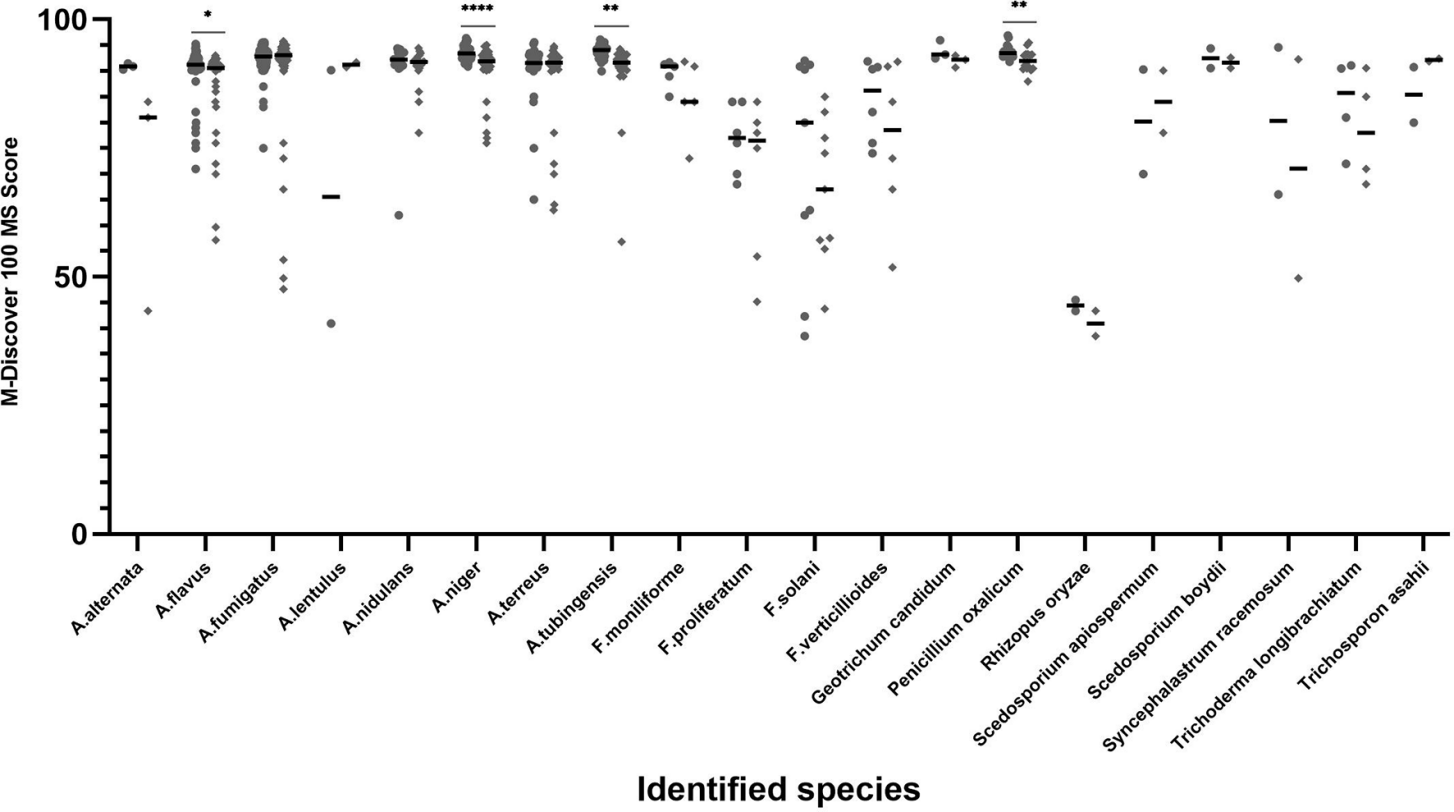
Average score and distribution by M-Discover 100 with the in-house library by method performed. Each dot represents the score for each isolate identification achieved by the ZSB (round dots) and routine (diamond dots) methods. Horizontal bars represent the mean identification score achieved by each method for each species including at least two isolates. A paired t-test was performed to analyze differences between the ZSB method and the routine method, and statistical significance was defined by *p* values less than 0.05 (*p < 0.05, **p < 0.01, ****p < 0.001).

The distribution and mean scores for each species that was isolated at least twice from individual clinical samples are shown in **Figure 2**. When coupled with the M-Discover 100 in-house library, the ZSB method showed comparable or higher maximum and mean scores for most species with the exception of *A. lentulus* and *Scedosporium apiospermum.* For the 20 species included, statistically significant differences were only observed in four species (*A. flavus*, *A. niger*, *A. tubinensis*, and *P. oxalicum*). From the point of the score classification, the species-level identification accuracy of strains with the identification score of ≥90 was 99.29% (419/422) provided by ZSB method and 99.49% (388/390) by the routine method. When the species-level cutoff value was artificially set to 60, the correct species-level identification rate was 98.47% (451/458) by ZSB and 98.20% (436/444) by routine method.

In addition, the use of the FUS method by the M-Discover 100 system simultaneously using the in-house library also showed good species-assignment of filamentous fungi (87.00%, 107/123), which was better than routine method (84.55%, 104/123), but inferior to the ZSB method (90.24%) (**Table S2**).

## Discussion

MALDI-TOF MS has gradually been popularized as an accurate, rapid and cost-effective method for routine identification of clinical filamentous fungi. However, unlike yeast and bacteria, routine clinical use for filamentous fungi is hindered by two main reasons: (i) insufficient filamentous fungi coverage in the commercial database (Schulthess et al., 2014; Li et al., 2017); (ii) prolonged testing time due to the multi-step protein extraction procedure.

To improve work efficiency, we created the one-step zirconia-silica beads method and focused-ultrasonication method for protein extraction. With the routine procedure, each filamentous fungi isolate required at least 30 min of sample processing before MALDI-TOF MS analysis (Luethy and Zelazny, 2018). The two optimized methods, however, negated the separate inactivation step without reducing the effect (no growth within 14 days after treated), while significantly reduced the dedicated operating time to 7 (ZSB) or 5 (FUS) min per sample, with only a few seconds added for each additional strain.

Of note, using the ZSB and FUS methods with the VITEK MS commercial database v3.2, all of the species resulted in comparable or better identification to the species level than the routine method, except for *A. nidulans*, *A. tubingensis*, and *Beauveria bassiana*. Both rapid methods can be utilized with the existing commercial database v3.2 in VITEK MS, without any required alterations of the database. Similar conclusions have been reported in another study by Luethy and Zelazny (2018). Their study evaluated the capacity of the zirconia-silica beads method combined the high-power bead-based homogenizer for the identification of molds using Bruker MALDI Biotyper, and reported more samples achieving clinically acceptable identification scores (≥2.00) than the routine method (63.0 *vs* 52.8%) (Adams et al., 2016; Luethy and Zelazny, 2018). Our optimized ZSB method provides significant cost savings in investment (no need for a homogenizer) compared to Luethy’s method without sacrificing identification effectiveness, while has been verified in two mass spectrometers. In addition, a previous study has proved that the FUS method can significantly increase the identification of mycobacteria by Bruker MALDI Biotyper (Adams et al., 2016). Combining the above two studies and our study, we believe our two rapid methods have universality in most mass spectrometer and species which identification hindered by difficulties related to peptide extraction due to the intrinsic characteristics of the cell. Future studies should be carried out to evaluate and optimize those methods for different mass spectrometers and for more species.

Although VITEK MS reliably identified various filamentous fungi by three methods, including *Aspergillus* and *Penicillium*, *Scedosporium*, and other species with a very low rate of misidentification that was not different from previous reports. For the *Fusarium* species, which tend to be multi-resistant and are the second most common filamentous fungi causing invasive fungal infections in immunocompromised patients, VITEK MS demonstrated a lower rate of correct identification to the species-level by all methods compared than to those in previous studies (100, 93.0, and 65.4%) (Heo et al., 2015; McMullen et al., 2016; Luethy and Zelazny, 2018; Rychert et al., 2018; Shin et al., 2021).

Given that all of the tested isolates in this study were from northern China, there may be intrinsic differences between the isolates included in the commercial database and those used in this study due to geographic variation (Adams et al., 2016). Specifically, *F. proliferatum* and *F. verticillioides* could not be distinguished by VITEK MS: further examination of the spectra obtained for these clinical isolates of two species revealed the closely related spectra pattern between them. Thus, it is necessary to increase the number of reference isolates in the database in order to distinguish between closely related species well (Lau et al., 2013).

On the other hand, we also evaluated the number of peaks and S/N ratios produced by three methods. Since the closed VITEK MS database, the spectra peak cannot be exported, we chose the peaks produced by the M-Discover 100 MS to spectrum analysis. Despite the less number of peaks generated, the two rapid methods generally achieved higher maximum and minimum S/N ratios with these isolates tested as compared to the routine method. It is worth noting that not all of these counted peaks are characteristic peaks identified by MS, thus this index may be not a good indicator of the quality of the extraction method. In addition, the ZSB method produced overall mean of maximum and minimum S/N ratio higher than that by FUS.

Evaluation of the rapid ZSB method not only revealed good applicability with the existing commercial database, but also demonstrated it can be a rapid and standardized protocol to construct an in-house library. In this study, we used the ZSB method as a protein extraction procedure to construct an in-house library in M-Discover 100 MS. The inclusion of 2,960 references from 42 species to our in-house database allowed the identification of 454 isolates at the species level (97.22%) using the ZSB method (**Table 3**), showing high correlation with DNA sequencing analysis regardless the score values. However, 10 isolates were identified only at the genus level and three completely misidentified, eleven of which were from four different species not available in the in-house database (*F. moniliforme*, *F. verticillioides*, *F. solani*, and *Rhizopus oryzae*). This highlights the necessity of adding endemic reference strains in the database in order to improve the identification capacity of MALDI-TOF MS. Besides, when lowering the species-level cutoff value to 60 in this study, the correct species-level identification rate showed a high robustness compared with that (≥90) in the manufacturer’s instructions (98.47 *vs* 99.29%). Further research can be done to establish appropriate cutoff value to improve the capacity of MALDI-TOF MS for filamentous fungi.

This in-house library can be used with other protein methods. Although the identification accuracy using the in-house library of both routine and FUS methods were inferior to the ZSB method, there were no statistically significant differences between them. However, the percent of isolates with scores ≥90 and 60–90 as generated by the routine method was lower than the ZSB method, while statistically significant differences were observed in four species (*A. flavus*, *A. niger*, *A. tubinensis*, and *P. oxalicum*) (**Figure 2**). There are several possibilities for why fewer accurate identifications occurred. First, it is plausible that the ZSB method breaks the cell wall thoroughly and can achieve higher protein content than the routine method. Further verification, such as comparing protein concentrations and profile are needed according to Akhila’s method (Krishnaswamy et al., 2019). Second, the spectra of the same strains had some differences produced by the ZSB and routine method, such as the number and relative position. Thus, the matching degree of the spectra by the routine method with the reference spectra by the ZSB method decreased slightly, resulting in lower scores.

This study has some limitations. First, the strains used in this study represent species typically encountered in northern China. And the *Aspergillus* species, along with *Fusarium* and *Penicillium* species, constitute over half of the tested isolates in this study. Thus, a more comprehensive list of filamentous fungi is needed and filamentous fungi species commonly encountered in other regions should be tested to further evaluate the two rapid methods. Second, the *Fusarium* species, the second most common strain in this study, was not added to the in-house library, while some common species had only one strain included in the in-house library. It is unknown whether geographic differences of the strains may result in variations in identification accuracy using the in-house library. Thus, updates to the M-Discover 100 in-house library are necessary to improve the identification ability.

## Conclusion

MALDI-TOF MS is widely used for filamentous fungi identification under the condition that an efficient sample processing procedure is implemented and an abundant library is available. Two rapid protein extraction methods we created for filamentous fungi isolates that not only significantly reduced sample processing time but also demonstrated superior maximum and minimum S/N ratio, and comparable or superior identification to the routine method when utilized with both the existing commercial database and the in-house library. Moreover, to our knowledge, this study represents the first implementation of the zirconia-silica beads method as the sample processing for building an in-house library in MALDI-TOF MS. We believe the advantages provided by the two rapid methods will attract more clinical laboratories to consider adopting MALDI-TOF MS for filamentous fungi identification.

## Data Availability Statement

The raw data supporting the conclusions of this article will be made available by the authors, without undue reservation.

## Ethics Statement

The program was approved by the Human Research Ethics Committee of Peking Union Medical College Hospital (S-263).

## Author Contributions

Y-TN processed the experimental data, performed the analysis, drafted the manuscript, and revised the manuscript. W-HY, M-X, W-Y designed the study and revised the manuscript. W-Z, J-JZ, GZ, and S-MD carried out the experiments, performed protein extraction and MALDI-TOF MS identification. A-YD, D-WG, G-LZ, HNW, Y-YG, L-P C, MC, J-DH, and QD contributed to strain collection, performed DNA sequencing identification. LZ and YCX revised the manuscript, and involved in planning and supervised the work. All authors contributed to the article and approved the submitted version.

## Funding

This work was supported by the National Major Science and Technology Project for the Control and Prevention of Major Infectious Diseases of China (nos. 2018ZX10712001 and 2020ZX1001015), Beijing Municipal Science and Technology Project (no. Z181100001618015), the National Natural Science Foundation of China (nos. 81802049 and 81971979), and Beijing Key Clinical Specialty for Laboratory Medicine - Excellent Project (no. ZK201000).

## Conflict of Interest

The authors declare that the research was conducted in the absence of any commercial or financial relationships that could be construed as a potential conflict of interest.
