# Supplementary material for: Developing Two Rapid Protein Extraction Methods Using Focused-Ultrasonication and Zirconia-Silica Beads for Filamentous Fungi Identification by MALDI-TOF MS
Source: Front Cell Infect Microbiol. 2021 Jul 6;11:687240. doi: 10.3389/fcimb.2021.687240 (PMC8290857; doi:10.3389/fcimb.2021.687240)
Supplement: Supplementary file 1 [file Table_1.docx]

Table S1. The number of mass peaks for 123 clinical filamentous fungi isolates

| Serial number | DNA sequencing results | The number of mass peaks | | |
| --- | --- | --- | --- | --- |
|  |  | Routine | ZSB | FUS |
| 19AA0590 | Alternaria alternata | 200 | 174 | 200 |
| 19AA0627 | Alternaria alternata | 200 | 78 | 91 |
| 19AA0895 | Alternaria alternata | 200 | 21 | 200 |
| 19AA0910 | Aspergillus flavus | 56 | 5 | 180 |
| 19AA0912 | Aspergillus flavus | 122 | 33 | 196 |
| 19AA0913 | Aspergillus flavus | 60 | 70 | 171 |
| 19AA0914 | Aspergillus flavus | 51 | 47 | 159 |
| 19AA0917 | Aspergillus flavus | 112 | 7 | 180 |
| 19AA0918 | Aspergillus flavus | 79 | 123 | 141 |
| 19AA0922 | Aspergillus flavus | 200 | 191 | 139 |
| 19AA0957 | Aspergillus flavus | 200 | 65 | 29 |
| 19AA0963 | Aspergillus flavus | 200 | 74 | 114 |
| 19AA0999 | Aspergillus flavus | 200 | 32 | 97 |
| 19AA0983 | Aspergillus fumigatus | 200 | 49 | 104 |
| 19AA0990 | Aspergillus fumigatus | 200 | 68 | 200 |
| 19AA0992 | Aspergillus fumigatus | 200 | 121 | 192 |
| 19AA0993 | Aspergillus fumigatus | 183 | 168 | 200 |
| 19AA0994 | Aspergillus fumigatus | 200 | 200 | 200 |
| 19AA0995 | Aspergillus fumigatus | 200 | 79 | 175 |
| 19AA0998 | Aspergillus fumigatus | 200 | 182 | 21 |
| 19AA1004 | Aspergillus fumigatus | 200 | 200 | 146 |
| 19AA1008 | Aspergillus fumigatus | 200 | 20 | 156 |
| 19AA1012 | Aspergillus fumigatus | 200 | 146 | 44 |
| 19AA0901 | Aspergillus lentulus | 96 | 17 | 54 |
| 19AA0944 | Aspergillus lentulus | 26 | 5 | 25 |
| 19AA0962 | Aspergillus lentulus | 39 | 200 | 200 |
| 19AA0657 | Aspergillus luchuensis | 200 | 104 | 96 |
| 19AA0329 | Aspergillus nidulans | 200 | 54 | 85 |
| 19AA0434 | Aspergillus nidulans | 196 | 88 | 137 |
| 19AA0727 | Aspergillus nidulans | 200 | 75 | 118 |
| 19AA0731 | Aspergillus nidulans | 200 | 157 | 200 |
| 19AA0753 | Aspergillus nidulans | 200 | 138 | 200 |
| 19AA0760 | Aspergillus nidulans | 200 | 112 | 145 |
| 19AA0779 | Aspergillus nidulans | 200 | 104 | 15 |
| 19AA0786 | Aspergillus nidulans | 163 | 108 | 79 |
| 19AA0814 | Aspergillus nidulans | 200 | 200 | 6 |
| 19AA0845 | Aspergillus nidulans | 190 | 60 | 75 |
| 19AA0511 | Aspergillus niger | 21 | 70 | 71 |
| 19AA0860 | Aspergillus niger | 189 | 52 | 154 |
| 19AA0863 | Aspergillus niger | 118 | 23 | 90 |
| 19AA0864 | Aspergillus niger | 200 | 193 | 126 |
| 19AA0871 | Aspergillus niger | 200 | 184 | 200 |
| 19AA0873 | Aspergillus niger | 125 | 153 | 173 |
| 19AA0886 | Aspergillus niger | 200 | 117 | 200 |
| 19AA0889 | Aspergillus niger | 139 | 36 | 77 |
| 19AA0892 | Aspergillus niger | 51 | 121 | 200 |
| 19AA0898 | Aspergillus niger | 18 | 90 | 96 |
| 19AA0437 | Aspergillus sydowii | 52 | 55 | 110 |
| 19AA0858 | Aspergillus terreus | 200 | 200 | 139 |
| 19AA0861 | Aspergillus terreus | 200 | 179 | 99 |
| 19AA0870 | Aspergillus terreus | 200 | 200 | 200 |
| 19AA0882 | Aspergillus terreus | 16 | 17 | 30 |
| 19AA0925 | Aspergillus terreus | 81 | 200 | 99 |
| 19AA0966 | Aspergillus terreus | 20 | 28 | 85 |
| 19AA0971 | Aspergillus terreus | 37 | 58 | 79 |
| 19AA0988 | Aspergillus terreus | 41 | 97 | 91 |
| 19AA0996 | Aspergillus terreus | 13 | 21 | 54 |
| 19AA1007 | Aspergillus terreus | 35 | 36 | 74 |
| 19AA0778 | Aspergillus tubingensis | 125 | 200 | 142 |
| 19AA0793 | Aspergillus tubingensis | 187 | 104 | 128 |
| 19AA0796 | Aspergillus tubingensis | 111 | 200 | 179 |
| 19AA0852 | Aspergillus tubingensis | 200 | 130 | 119 |
| 19AA0853 | Aspergillus tubingensis | 68 | 99 | 122 |
| 19AA0888 | Aspergillus tubingensis | 40 | 200 | 101 |
| 19AA0890 | Aspergillus tubingensis | 135 | 200 | 200 |
| 19AA0905 | Aspergillus tubingensis | 171 | 127 | 117 |
| 19AA0923 | Aspergillus tubingensis | 200 | 200 | 192 |
| 19AA0929 | Aspergillus tubingensis | 200 | 200 | 200 |
| 19AA0835 | Aspergillus ustus | 200 | 28 | 186 |
| 19AA0757 | Beauveria bassiana | 80 | 38 | 53 |
| 19AA0689 | Fusarium incarnatum | 200 | 100 | 67 |
| 19AA0520 | Fusarium proliferatum | 200 | 84 | 30 |
| 19AA0591 | Fusarium proliferatum | 200 | 83 | 133 |
| 19AA0621 | Fusarium proliferatum | 200 | 50 | 50 |
| 19AA0692 | Fusarium proliferatum | 166 | 21 | 22 |
| 19AA0812 | Fusarium proliferatum | 200 | 20 | 11 |
| 19AA0894 | Fusarium proliferatum | 63 | 139 | 200 |
| 19AA0581 | Fusarium solani | 73 | 41 | 20 |
| 19AA0584 | Fusarium solani | 200 | 164 | 200 |
| 19AA0620 | Fusarium solani | 200 | 73 | 120 |
| 19AA0694 | Fusarium solani | 80 | 91 | 103 |
| 19AA0695 | Fusarium solani | 173 | 74 | 65 |
| 19AA0883 | Fusarium solani | 181 | 129 | 134 |
| 19AA0972 | Fusarium solani | 175 | 33 | 123 |
| 19AA0984 | Fusarium solani | 185 | 200 | 28 |
| 19AA1011 | Fusarium solani | 170 | 153 | 157 |
| 19AA0522 | Fusarium verticillioides | 12 | 89 | 102 |
| 19AA0531 | Fusarium verticillioides | 200 | 114 | 117 |
| 19AA0612 | Fusarium verticillioides | 101 | 6 | 25 |
| 19AA0623 | Fusarium verticillioides | 68 | 33 | 50 |
| 19AA0846 | Fusarium verticillioides | 200 | 135 | 97 |
| 19AA0897 | Fusarium verticillioides | 92 | 41 | 76 |
| 19AA0534 | Geotrichum candidum | 200 | 46 | 32 |
| 19AA0652 | Geotrichum candidum | 200 | 12 | 26 |
| 19AA0969 | Geotrichum candidum | 200 | 101 | 69 |
| 19AA0752 | Mucor circinelloides | 200 | 74 | 24 |
| 19AA0859 | Penicillium chrysogenum | 82 | 48 | 60 |
| 19AA0521 | Penicillium citrinum | 30 | 68 | 48 |
| 19AA0483 | Penicillium oxalicum | 200 | 55 | 33 |
| 19AA0486 | Penicillium oxalicum | 177 | 24 | 22 |
| 19AA0602 | Penicillium oxalicum | 70 | 61 | 50 |
| 19AA0604 | Penicillium oxalicum | 46 | 5 | 52 |
| 19AA0849 | Penicillium oxalicum | 109 | 80 | 31 |
| 19AA0850 | Penicillium oxalicum | 125 | 49 | 40 |
| 19AA0896 | Penicillium oxalicum | 70 | 18 | 30 |
| 19AA0946 | Penicillium oxalicum | 151 | 94 | 56 |
| 19AA0976 | Penicillium oxalicum | 200 | 99 | 117 |
| 19AA1001 | Penicillium oxalicum | 200 | 14 | 132 |
| 19AA0420 | Rhizopus oryzae | 39 | 53 | 54 |
| 19AA0908 | Rhizopus oryzae | 162 | 42 | 34 |
| 19AA0804 | Scedosporium apiospermum | 200 | 19 | 12 |
| 19AA0826 | Scedosporium apiospermum | 83 | 20 | 42 |
| 19AA0680 | Scedosporium aurantiacum | 200 | 58 | 34 |
| 19AA0519 | Scedosporium boydii | 96 | 114 | 178 |
| 19AA0696 | Scedosporium boydii | 200 | 29 | 86 |
| 19AA0874 | Scopulariopsis brevicaulis | 200 | 103 | 200 |
| 19AA0499 | Sporothrix schenckii | 162 | 34 | 103 |
| 19AA0474 | Syncephalastrum racemosum | 109 | 39 | 34 |
| 19AA0713 | Syncephalastrum racemosum | 85 | 83 | 43 |
| 19AA0400 | Trichoderma longibrachiatum | 97 | 30 | 45 |
| 19AA0418 | Trichoderma longibrachiatum | 200 | 26 | 31 |
| 19AA0920 | Trichoderma longibrachiatum | 200 | 33 | 35 |
| 19AA0951 | Trichoderma longibrachiatum | 102 | 63 | 38 |

**Table S2.** Identification of 123 clinical filamentous fungi isolates by M-Disocver 100 with in-house library using the three methods.

| **Identification by DNA sequencing analysis** | | **Number** | **Routine method** | | |  | **ZSB method** | | |  | **FUS method** | | |
| --- | --- | --- | --- | --- | --- | --- | --- | --- | --- | --- | --- | --- | --- |
|  |  |  | Number of isolates | | |  | Number of isolates | | |  | Number of isolates | | |
|  |  |  | Specie level | Genus level | Mis-ID |  | Specie level | Genus level | Mis-ID |  | Specie level | Genus level | Mis-ID |
| ***Aspergillus*** | |  |  |  |  |  |  |  |  |  |  |  |  |
|  | *A. flavus* | 10 | 10 | 0 | 0 |  | 10 | 0 | 0 |  | 10 | 0 | 0 |
|  | *A. fumigatus* | 10 | 9 | 1 | 0 |  | 10 | 0 | 0 |  | 10 | 0 | 0 |
|  | *A. lentulus* | 3 | 2 | 1 | 0 |  | 2 | 1 | 0 |  | 1 | 1 | 1 |
|  | *A. luchuensis* | 1 | 1 | 0 | 0 |  | 1 | 0 | 0 |  | 1 | 0 | 0 |
|  | *A. nidulans* | 10 | 10 | 0 | 0 |  | 10 | 0 | 0 |  | 10 | 0 | 0 |
|  | *A. niger* | 10 | 10 | 0 | 0 |  | 10 | 0 | 0 |  | 10 | 0 | 0 |
|  | *A. sydowii* | 1 | 1 | 0 | 0 |  | 1 | 0 | 0 |  | 1 | 0 | 0 |
|  | *A. terreus* | 10 | 10 | 0 | 0 |  | 10 | 0 | 0 |  | 10 | 0 | 0 |
|  | 1. *tubingensis* | 10 | 10 | 0 | 0 |  | 9 | 1 | 0 |  | 10 | 0 | 0 |
|  | 1. *ustus* | 1 | 1 | 0 | 0 |  | 1 | 0 | 0 |  | 0 | 1 | 0 |
|  | **Subtotal** | **66** | **64** | **2** | **0** |  | **64** | **2** | **0** |  | **63** | **2** | **1** |
| ***Fusarium*** | |  |  |  |  |  |  |  |  |  |  |  |  |
|  | *F. incarnatum* | 1 | 1 | 0 | 0 |  | 1 | 0 | 0 |  | 1 | 0 | 0 |
|  | *F. proliferatum* | 6 | 4 | 1 | 1 |  | 6 | 0 | 0 |  | 5 | 1 | 0 |
|  | *F. solani* | 9 | 8 | 0 | 1 |  | 7 | 0 | 2 |  | 8 | 0 | 1 |
|  | *F. verticillioides* | 6 | 0 | 6 | 0 |  | 0 | 6 | 0 |  | 0 | 6 | 0 |
|  | **Subtotal** | **22** | **13** | **7** | **2** |  | **14** | **6** | **2** |  | **14** | **7** | **1** |
| ***Penicillium*** | |  |  |  |  |  |  |  |  |  |  |  |  |
|  | 1. *chrysogenum* | 1 | 1 | 0 | 0 |  | 1 | 0 | 0 |  | 0 | 0 | 1 |
|  | *P. citrinum* | 1 | 1 | 0 | 0 |  | 1 | 0 | 0 |  | 1 | 0 | 0 |
|  | *P. oxalicum* | 10 | 10 | 0 | 0 |  | 10 | 0 | 0 |  | 9 | 0 | 1 |
|  | **Subtotal** | **12** | **12** | **0** | **0** |  | **12** | **0** | **0** |  | **10** | **0** | **2** |
| ***Scedosporium*** | |  |  |  |  |  |  |  |  |  |  |  |  |
|  | *S. apiospermum* | 2 | 2 | 0 | 0 |  | 2 | 0 | 0 |  | 2 | 0 | 0 |
|  | *S. aurantiacum* | 1 | 1 | 0 | 0 |  | 1 | 0 | 0 |  | 1 | 0 | 0 |
|  | *S. boydii* | 2 | 2 | 0 | 0 |  | 2 | 0 | 0 |  | 2 | 0 | 0 |
|  | **Subtotal** | **5** | **5** | **0** | **0** |  | **5** | **0** | **0** |  | **5** | **0** | **0** |
| **Others** | |  |  |  |  |  |  |  |  |  |  |  |  |
|  | *Alternaria alternata* | 3 | 2 | 0 | 1 |  | 3 | 0 | 0 |  | 3 | 0 | 0 |
|  | *Beauveria bassiana* | 1 | 0 | 0 | 1 |  | 1 | 0 | 0 |  | 1 | 0 | 0 |
|  | *Geotrichum candidum* | 3 | 3 | 0 | 0 |  | 3 | 0 | 0 |  | 3 | 0 | 0 |
|  | *Mucor circinelloides* | 1 | 1 | 0 | 0 |  | 1 | 0 | 0 |  | 0 | 0 | 1 |
|  | *Rhizopus oryzae* | 2 | 0 | 0 | 2 |  | 0 | 1 | 1 |  | 0 | 1 | 1 |
|  | *Scopulariopsis brevicaulis* | 1 | 1 | 0 | 0 |  | 1 | 0 | 0 |  | 1 | 0 | 0 |
|  | *Sporothrix schenckii* | 1 | 1 | 0 | 0 |  | 1 | 0 | 0 |  | 1 | 0 | 0 |
|  | *Syncephalastrum racemosum* | 2 | 1 | 0 | 1 |  | 2 | 0 | 0 |  | 2 | 0 | 0 |
|  | *Trichoderma longibrachiatum* | 4 | 1 | 3 | 0 |  | 4 | 0 | 0 |  | 4 | 0 | 0 |
|  | **Subtotal** | **18** | **10** | **3** | **5** |  | **16** | **1** | **1** |  | **15** | **1** | **2** |
| **Total** | | **123** | **104** | **12** | **7** |  | **111** | **9** | **3** |  | **107** | **10** | **6** |
